# Feasibility of Artificial Intelligence–Based Electrocardiography Analysis for the Prediction of Obstructive Coronary Artery Disease in Patients With Stable Angina: Validation Study

**DOI:** 10.2196/44791

**Published:** 2023-05-02

**Authors:** Jiesuck Park, Yeonyee Yoon, Youngjin Cho, Joonghee Kim

**Affiliations:** 1 Department of Cardiology Seoul National University Bundang Hospital Seongnam, Gyeonggi-do Republic of Korea; 2 Department of Emergency Medicine, Seoul National University Bundang Hospital Seongnam, Gyeonggi-do Republic of Korea

**Keywords:** artificial intelligence, AI, coronary artery disease, coronary stenosis, electrocardiography, stable angina

## Abstract

**Background:**

Despite accumulating research on artificial intelligence–based electrocardiography (ECG) algorithms for predicting acute coronary syndrome (ACS), their application in stable angina is not well evaluated.

**Objective:**

We evaluated the utility of an existing artificial intelligence–based quantitative electrocardiography (QCG) analyzer in stable angina and developed a new ECG biomarker more suitable for stable angina.

**Methods:**

This single-center study comprised consecutive patients with stable angina. The independent and incremental value of QCG scores for coronary artery disease (CAD)–related conditions (ACS, myocardial injury, critical status, ST-elevation myocardial infarction, and left ventricular dysfunction) for predicting obstructive CAD confirmed by invasive angiography was examined. Additionally, ECG signals extracted by the QCG analyzer were used as input to develop a new QCG score.

**Results:**

Among 723 patients with stable angina (median age 68 years; male: 470/723, 65%), 497 (69%) had obstructive CAD. QCG scores for ACS and myocardial injury were independently associated with obstructive CAD (odds ratio [OR] 1.09, 95% CI 1.03-1.17 and OR 1.08, 95% CI 1.02-1.16 per 10-point increase, respectively) but did not significantly improve prediction performance compared to clinical features. However, our new QCG score demonstrated better prediction performance for obstructive CAD (area under the receiver operating characteristic curve 0.802) than the original QCG scores, with incremental predictive value in combination with clinical features (area under the receiver operating characteristic curve 0.827 vs 0.730; *P*<.001).

**Conclusions:**

QCG scores developed for acute conditions show limited performance in identifying obstructive CAD in stable angina. However, improvement in the QCG analyzer, through training on comprehensive ECG signals in patients with stable angina, is feasible.

## Introduction

Coronary artery disease (CAD) is a major global health issue, with increasing prevalence and incidence worldwide [1]. Although electrocardiography (ECG) has been used as a primary noninvasive modality in patients with suspected CAD, its diagnostic value is limited to acute coronary syndrome (ACS), such as ST-elevation myocardial infarction (STEMI) [2]. In patients presenting with stable angina, the initial ECG often shows nonspecific or normal findings, resulting in a low-diagnostic accuracy [2,3]. Therefore, in most cases, additional tests, such as exercise ECG, single-photon emission computed tomography, and coronary computed tomography angiography, are required regardless of the initial ECG findings.

In previous decades, several attempts have been made to develop automated algorithms using artificial intelligence (AI) to analyze ECG signals to identify CAD [4]. For clinical use, most of these AI-ECG “digital biomarkers” were developed for the rapid evaluation of patients presenting with acute chest pain, especially for ACS screening [5-10]. However, for nonacute conditions, there is a paucity of data regarding the use of such AI algorithms. Accordingly, there is a large knowledge gap regarding whether algorithms developed for acute ischemia can be used in chronic stable conditions.

We hypothesized that the AI-ECG biomarkers originally developed for acute ischemia might also provide valuable information regarding the presence and severity of CAD in patients presenting with stable angina. However, we also considered the possibility that a dedicated score for stable angina may be more suitable. Therefore, the objectives of this study were 2-fold: (1) to evaluate the utility of ECG digital biomarkers that were originally developed for acute conditions, such as ACS, in the risk stratification of patients presenting with stable angina; and (2) to evaluate the feasibility of developing a new ECG biomarker for stable angina by reusing the deep features of an existing AI system.

## Methods

### Study Population

We retrospectively screened consecutive patients who visited the outpatient clinic with symptoms indicative of stable angina and underwent invasive coronary angiography at the Seoul National University Bundang Hospital (SNUBH) between 2018 and 2020. Symptomatic patients with suspected CAD and available records on clinical risk factors and baseline examinations (blood tests, chest X-ray, ECG, and echocardiography) were included. Patients who were asymptomatic, had a previous history of CAD or coronary revascularization (including percutaneous coronary intervention and bypass surgery), underwent emergent or urgent coronary angiography with suspected ACS, or underwent an ergonovine provocation test with suspected variant angina were excluded. Finally, 723 patients were analyzed.

### Ethics Approval

The study protocol was approved by the institutional review board of SNUBH (B-2211-790-102) and conducted in compliance with the principles of the Declaration of Helsinki. The requirement for informed consent was waived by the review board due to the retrospective study design.

### Clinical Features and Invasive Coronary Angiography

Baseline characteristics were obtained through a dedicated review of electronic health records. Patient symptoms were classified as typical or atypical chest pain according to their nature. Additionally, patients without documented chest pain but with relevant symptoms indicative of ischemic heart disease (eg, dyspnea, diaphoresis, or extreme fatigue) were categorized as having angina-equivalent symptoms. The presence of clinical risk factors, including hypertension, diabetes mellitus, dyslipidemia, and stroke, were determined by clinical diagnoses or medical therapy records.

All patients underwent invasive coronary angiography, and obstructive CAD was defined as the presence of any stenosis with ≥50% diameter stenosis in major epicardial coronary arteries (left main [LM], left anterior descending artery, left circumflex artery, or right coronary artery [RCA]), or major branches of each artery. The number of vessels with obstructive CAD was counted as 1, 2, or 3 (1 vessel with obstructive CAD [VD], 2VD, or 3VD), and obstructive CAD at the LM was considered equivalent to 2VD. Therefore, 3VD was defined as the presence of obstructive CAD in all 3 epicardial coronary arteries (left anterior descending artery, left circumflex artery, and RCA) or in both the LM coronary artery and RCA.

### AI-Based Quantitative Electrocardiography (QCG) Analysis

**Figure 1:**
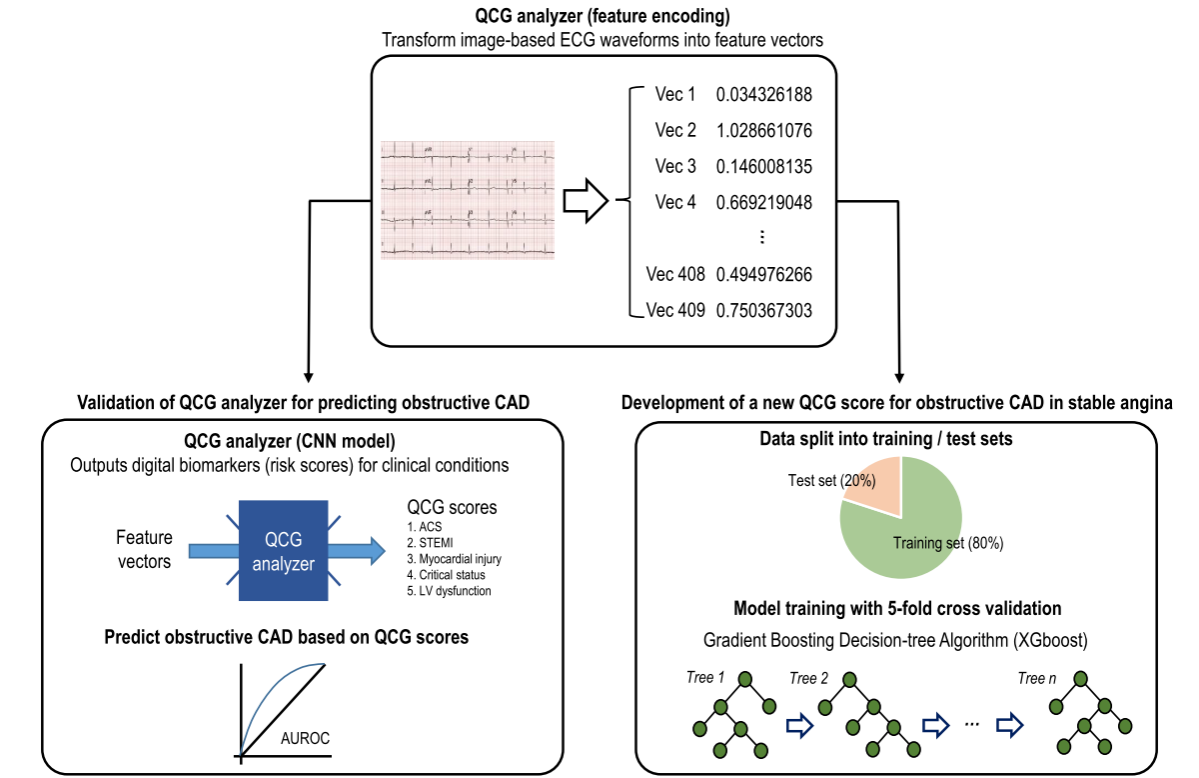
Artificial intelligence (AI)–based quantitative electrocardiography (QCG) analysis. The diagram summarizes the study flow. The QCG analyzer transforms image-based electrocardiography (ECG) data into vectorized signal features. To validate the original QCG analyzer in patients with stable angina, we derived 5 QCG scores related to coronary artery disease (CAD) and evaluated their predictive value for the presence of obstructive CAD. Additionally, we developed a new QCG score that is more suitable for identifying obstructive CAD in stable angina by using a tree-based AI algorithm, XGboost, with the extracted vectors as input features. ACS: acute coronary syndrome; AUROC: area under the receiver operating characteristic curve; CNN: convolutional neural network; LV: left ventricular; STEMI: ST-elevation myocardial infarction.

The development process for the QCG analyzer (Figure 1), which operates on a mobile platform as a smartphone app, has been reported previously [5,6]. The QCG analyzer is composed of an encoder and various task-specific artificial neural network layers. The encoder is a deep learning algorithm pretrained on various open ECG data sets (49,731 recordings total) using self-supervised learning techniques and can be used with or without fine-tuning for various tasks. It was first integrated into a smartphone AI app developed to screen various emergency conditions using 47,194 annotated ECG images of over 32,968 patients who were admitted to the SNUBH emergency department between 2017 and 2019. The encoder part has a signal extraction submodule applying a series of morphological operation procedures on the input data and a ResNet-based convolutional neural network submodule with 16 layers of convolution layers with squeeze-excitation blocks and a nonlocal network block. The encoder serves as a signal extractor and both submodules can be jointly optimized further through fine tuning for the specific tasks it is applied for. The task-specific layers of the QCG analyzer were optimized with Adam optimizer with focal loss function using a supervised training scheme, and the output probability from the sigmoid function was calibrated using the temperature scaling method. The QCG analyzer outputs 10 digital biomarkers (QCG scores ranging from 0 to 100) representing the risk of 10 medical conditions that may require emergent management (ACS, STEMI, myocardial injury, critical status, left ventricular [LV] dysfunction, pulmonary edema, pulmonary hypertension, right ventricular dysfunction, pericardial effusion, and hyperkalemia).

In this study, the QCG analyzer was used in the following 2 AI-ECG analyses (Figure 1). First, to evaluate whether the original QCG analyzer trained on acute-phase patients admitted to the emergency department could be used for the evaluation of patients presenting with stable angina, we derived 5 QCG scores related to CAD (ACS, STEMI, myocardial injury, critical status, and LV dysfunction). We then examined the independent association and predictive value of each QCG score for the presence of (1) any obstructive CAD and (2) 3VD as study outcomes. Second, to develop a new QCG score for the detection of any obstructive CAD that is more suitable for stable angina, we extracted the encoded feature vectors from input ECG images using the QCG analyzer, which were then used as input features.

### Statistical Analysis

All statistical analyses were performed using R software (version 4.2.1; R Core Team). Continuous variables were presented as medians (IQR; categorical variables were presented as numbers (percentages). A 2-sided *P*<.05 was considered statistically significant.

### Evaluation of Original QCG Scores for Predicting Obstructive CAD in Patients With Stable Angina

QCG scores for 5 CAD-related conditions were plotted according to the number of vessels affected to evaluate their distribution according to the CAD burden in patients with stable angina. Significant associations between QCG scores and study outcomes were examined using logistic regression analysis, with independence determined by multivariate adjustment for clinical features. The discrimination performance of QCG scores for the study outcomes was evaluated by the area under the receiver operating characteristic curve (AUROC). To evaluate the incremental predictive value of QCG score over clinical features, we constructed the clinical model using multivariate logistic regression analysis with baseline clinical variables (age, sex, BMI, symptom type, hypertension, diabetes mellitus, dyslipidemia, smoking, stroke, and family history of cardiovascular disease). Compared with the baseline performance of the clinical model, significant improvement in AUROC was assessed by adding QCG scores with clinical features in the prediction model.

### Development of a New Risk Score for Obstructive CAD in Stable Angina

To derive a new QCG score suitable for stable angina, the entire data set was divided into a training set (80%) and a test set (20%). For model development, we employed an existing AI algorithm using a tree-based ensemble technique, XGboost, which has shown satisfactory performance in disease classification problems across various cardiovascular fields [11]. The final prediction model was derived from the training set with hyperparameter optimization. We applied a 5-fold cross-validation technique for this hyperparameter tuning process by randomly dividing the training set into 5 folds. During the cross-validation, the model was fitted among the 4 folds and validated on the remaining fold, and this step was repeated 5 times. Then the final model performance on outcome prediction was verified in the test set, expressed as AUROC. Additionally, the incremental predictive value of the new QCG score was assessed among the test set. The baseline clinical model was constructed with clinical features that were the same as those used for assessing the incremental value of the original QCG score.

## Results

### Baseline Clinical Features

The clinical features of 723 patients (age: median 68 years, IQR 60-75 years; male: 470/723, 65%; BMI 25.1 kg/m^2^, IQR 23.3-27.1 kg/m^2^) are summarized in Table 1. Obstructive CAD was found in 497 (69%) patients, 132 (18%) of whom had 3VD. Patients with obstructive CAD tended to be male, presented more frequently with typical chest pain, and had a higher prevalence of clinical risk factors than those without obstructive CAD. These trends were more pronounced in patients with 3VD (Table 1).

**Table 1:** Baseline characteristics.

	Variables			Total population (N=723)		No obstructive CAD^a^ (n=226)			Any obstructive CAD (n=497)		*P* value (vs no obstructive CAD)		3-vessel obstructive CAD (n=132)		*P* value (vs no obstructive CAD)
**Demographics**															
	Age (years), median (IQR)			68 (60- 75)		68 (59-74)		68 (60-75)			.39		69 (61-76)		.28
	Male, n (%)			470 (65)		125 (55.3)		345 (69.4)			<.001		98 (74.2)		<.001
	BMI (kg/m^2^), median (IQR)			25.1 (23.3-27.1)		25.3 (23.2-27.5)		25.1 (23.4-27.1)			.46		25.3 (23.6-27.1)		.90
**Clinical features, n (%)**															
	**Symptoms**														
		Typical chest pain	486 (67.2)		125 (55.3)		361 (72.6)			<.001		111 (84.1)		<.001	
		Atypical chest pain	163 (22.5)		70 (31)		93 (18.7)			—^b^		12 (9.1)		—	
		Angina equivalent	74 (10.2)		31 (13.7)		43 (8.7)			—		9 (6.8)		—	
	Hypertension			459 (63.5)		130 (57.5)		329 (66.2)			.03		98 (74.2)		.002
	Diabetes mellitus			221 (30.6)		55 (24.3)		166 (33.4)			.02		55 (41.7)		.001
	Dyslipidemia			245 (33.9)		71 (31.4)		174 (35)			.39		51 (38.6)		.20
	Stroke			41 (5.7)		8 (3.5)		33 (6.6)			.13		9 (6.8)		.25
	Family history of CAD			59 (8.2)		10 (4.4)		49 (9.9)			.02		10 (7.6)		.31
	Smoking			68 (9.4)		19 (8.4)		49 (9.9)			.63		13 (9.8)		.79
**Invasive coronary angiography, n (%)**															
	**Obstructive CAD**														
		Left main	56 (7.7)		—		56 (11.3)			—		38 (28.8)		—	
		Left anterior descending artery	402 (55.6)		—		402 (80.9)			—		125 (94.7)		—	
		Left circumflex artery	218 (30.2)		—		218 (43.9)			—		119 (90.2)		—	
		Right coronary artery.	248 (34.3)		—		248 (49.9)			—		132 (100)		—	

^a^CAD: coronary artery disease.

^b^Not applicable.

### Distribution of the Original QCS Scores in Patients Presenting With Stable Angina

**Figure 2:**
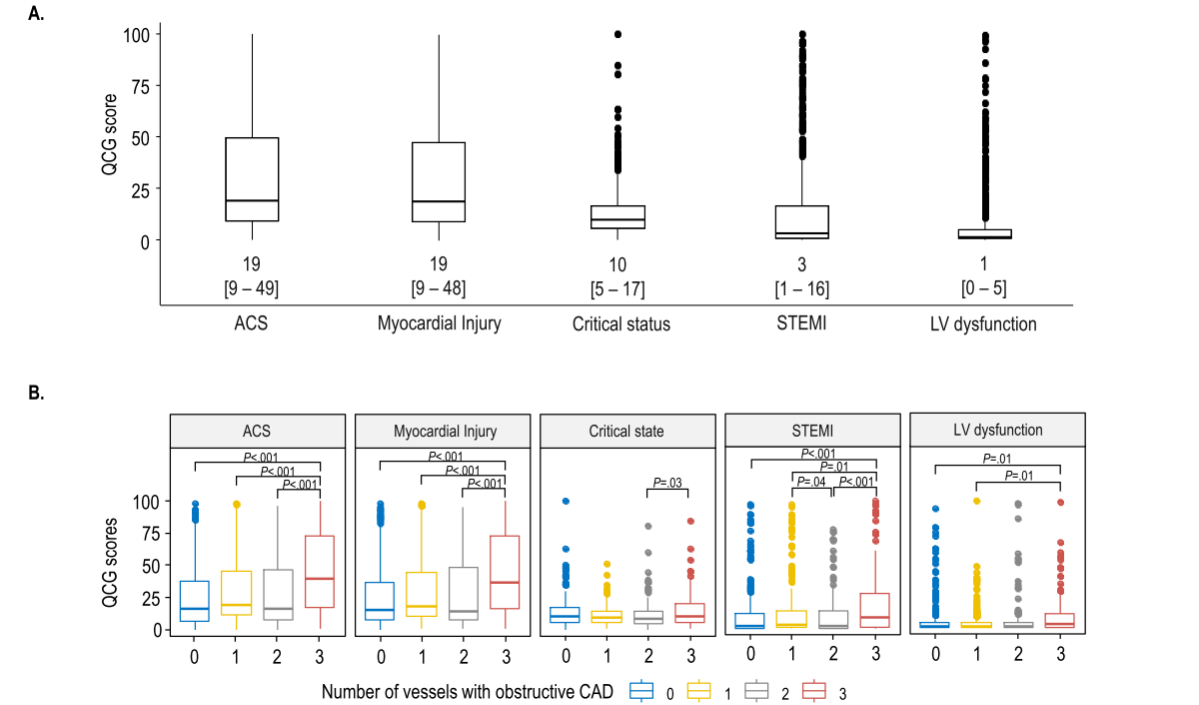
Distribution of the quantitative electrocardiography (QCG) scores. (A) Among the 5 QCG scores, those for acute coronary syndrome (ACS) and myocardial injury exhibited the highest values. (B) When the QCG scores were stratified by the number of vessels with obstructive coronary artery disease (CAD), patients group with 3-vessel disease showed significantly higher scores for ACS and myocardial injury than those in the other groups. LV: left ventricular; STEMI: ST-elevation myocardial infarction.

The distributions of 5 QCG scores in the study population are shown in Figure 2. Among these, the scores for ACS (median 19, IQR 9-49) and myocardial injury (median 19, IQR 9-48) had the highest values (Figure 2A). When stratified by the number of vessels with obstructive CAD, these 2 scores did not significantly differ between patients with 1VD or 2VD and those without obstructive CAD (Figure 2B). However, the 3VD group demonstrated significantly higher ACS and myocardial injury QCG scores compared to those in other groups (all *P*<.01).

### Predictive Value of the Original QCG Scores

On univariate analysis, only the QCG scores for ACS (odds ratio [OR] 1.11, 95% CI 1.05-1.18 per 10-point increase; *P*<.001) and myocardial injury (OR 1.10, 95% CI 1.04-1.17 per 10-point increase; *P*=.002) showed significant associations with the presence of obstructive CAD (Table 2). However, all 5 QCG scores demonstrated significant associations with 3VD. These trends were maintained after multivariate adjustment for clinical features (age, sex, BMI, type of chest pain, hypertension, diabetes, dyslipidemia, smoking, stroke, and family history of premature CAD). Only QCG scores for ACS and myocardial injury showed independent associations with the presence of obstructive CAD (OR 1.09, 95% CI 1.03-1.17 per 10-point increase; *P*=.006; and OR 1.08, 95% CI 1.02-1.16 per 10-point increase; *P*=.01; respectively) (Table 3); however, all 5 QCG scores were independently associated with the presence of 3VD. The QCG scores for ACS (OR 1.19, 95% CI 1.11-1.27 per 10-point increase; *P*<.001) and myocardial injury (OR 1.19, 95% CI 1.10-1.26 per 10-point increase; *P*<.001) demonstrated stronger associations with 3VD than other QCG scores (Table 3).

**Figure 3:**
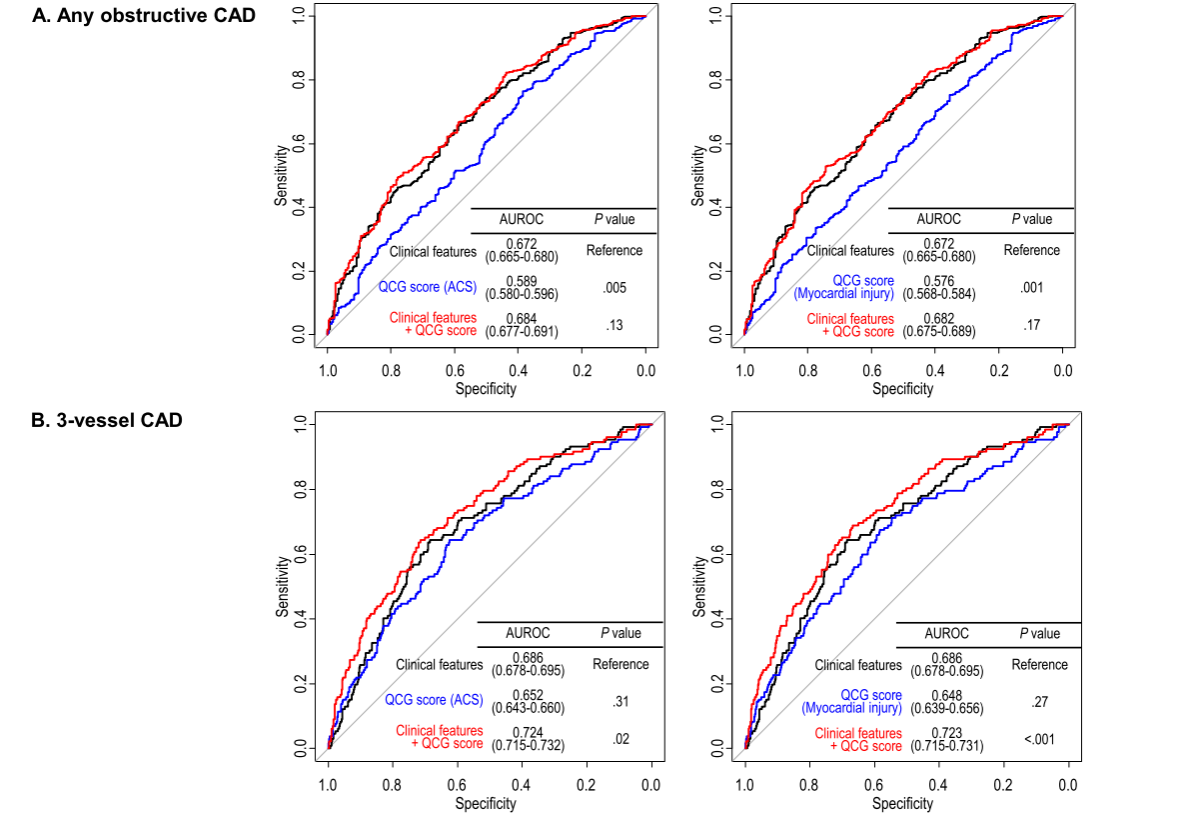
Incremental predictive value of the original quantitative electrocardiography (QCG) scores for obstructive coronary artery disease (CAD). (A) The QCG scores for acute coronary syndrome (ACS) and myocardial injury demonstrated significant predictive performance for obstructive CAD, despite lower performance than that of clinical features. (B) In contrast, both scores showed moderate predictive performance for 3-vessel disease, comparable to that for clinical features, and provide incremental predictive value in combination with clinical features. AUROC: area under the receiver operating characteristic curve.

Although both scores for ACS and myocardial injury showed significant discriminative performance for obstructive CAD (AUROC 0.589 and 0.576, respectively), their performance was lower than that for clinical features (AUROC 0.672; *P*=.005 and *P*=.001, respectively) (Figure 3A). However, for the prediction of 3VD, both scores demonstrated moderate performance (AUROC 0.652 and 0.648, respectively), comparable to that for clinical features (AUROC 0.686; *P*=.31 and *P*=.27, respectively), and provided significant incremental predictive value in combination with clinical features (AUROC 0.724 and 0.723; *P*=.02 and *P*<.001, respectively) (Figure 3B).

**Table 2:** Univariate analysis for obstructive coronary artery disease (CAD).

Variables			Any obstructive CAD				3-vessel obstructive CAD		
			Univariate OR^a^ (95% CI)		*P* value		Univariate OR (95% CI)		*P* value
**Clinical features**									
	Age (per 10-year increase)	1.10 (0.95-1.27)		.20		1.10 (0.92-1.31)		.29	
	Male	1.83 (1.33-2.54)		<.001		1.70 (1.11-2.59)		.02	
	BMI (per 1 kg/m^2^ increase)	0.97 (0.92-1.01)		.15		1.00 (0.94-1.06)		.99	
	Typical chest pain	2.14 (1.54-2.98)		<.001		3.04 (1.85-5.00)		<.001	
	Hypertension	1.45 (1.05-2.00)		.03		1.84 (1.20-2.81)		.005	
	Diabetes mellitus	1.56 (1.09-2.23)		.02		1.83 (1.24-2.70)		.002	
	Dyslipidemia	1.18 (0.84-1.65)		.34		1.29 (0.87-1.90)		.20	
	Stroke	1.94 (0.88-4.27)		.10		1.28 (0.59-2.75)		.53	
	Family history of CAD	2.36 (1.17-4.75)		.02		0.91 (0.45-1.84)		.79	
	Smoking	1.19 (0.68-2.08)		.54		1.06 (0.56-2.01)		.85	
**QCG^b^** **scores (per 10-point increase)**									
	Acute coronary syndrome	1.11 (1.05-1.18)		<.001		1.20 (1.12-1.27)		<.001	
	Myocardial injury	1.10 (1.04-1.17)		.002		1.19 (1.12-1.27)		<.001	
	Critical status	0.94 (0.82-1.08)		.40		1.21 (1.04-1.41)		.02	
	STEMI^c^	1.04 (0.97-1.12)		.30		1.13 (1.05-1.21)		.002	
	Left ventricle dysfunction	0.98 (0.88-1.09)		.71		1.14 (1.02-1.27)		.02	

^a^OR: odds ratio.

^b^QCG: quantitative electrocardiography.

^c^STEMI: ST-elevation myocardial infarction.

**Table 3:** Multivariate analysis of the quantitative electrocardiography (QCG) scores for obstructive coronary artery disease (CAD).

Variables			Any obstructive CAD					3-vessel obstructive CAD		
			Multivariate OR (95% CI)^a^		*P* value		Multivariate OR (95% CI)			*P* value
**QCG scores (per 10-point increase)**										
	Acute coronary syndrome	1.09 (1.03-1.17)		.006		1.19 (1.11-1.27)			<.001	
	Myocardial injury	1.08 (1.02-1.16)		.01		1.19 (1.10-1.26)			<.001	
	Critical status	0.92 (0.79-1.06)		.24		1.19 (1.01-1.40)			.04	
	STEMI^b^	1.03 (0.95-1.11)		.52		1.13 (1.04-1.22)			.004	
	Left ventricle dysfunction	0.95 (0.85-1.06)		.37		1.13 (1.01-1.27)			.04	

^a^Multivariate odds ratios (ORs) are estimated with adjustment for age, sex, body mass index, typical symptoms, hypertension, diabetes mellitus, dyslipidemia, stroke, family history of CAD, and smoking.

^b^STEMI: ST-elevation myocardial infarction.

### A New QCG Score for Obstructive CAD in Stable Angina

**Figure 4:**
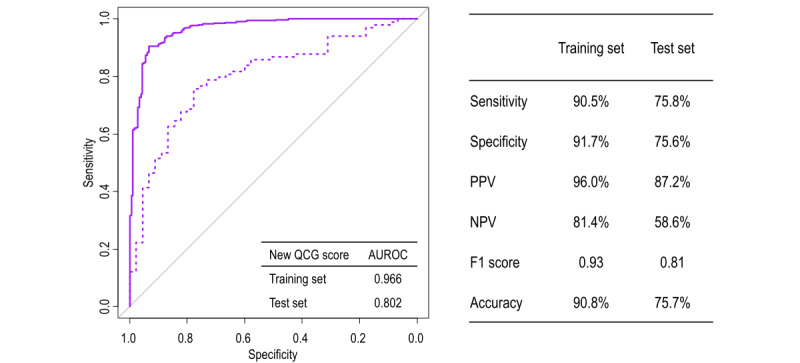
Prediction performance of the new quantitative electrocardiography (QCG) score for any obstructive coronary artery disease (CAD). This figure summarizes the discrimination performance of the new QCG score in the training and test sets. After categorizing the patients by the optimal cutoff values of the new QCG score (score of 66 in both training and test sets), the accuracy for obstructive CAD was 90.8% and 75.7% in the training and test sets, respectively. AUROC: area under the receiver operating characteristic curve; NPV: negative predictive value; PPV: positive predictive power.

**Figure 5:**
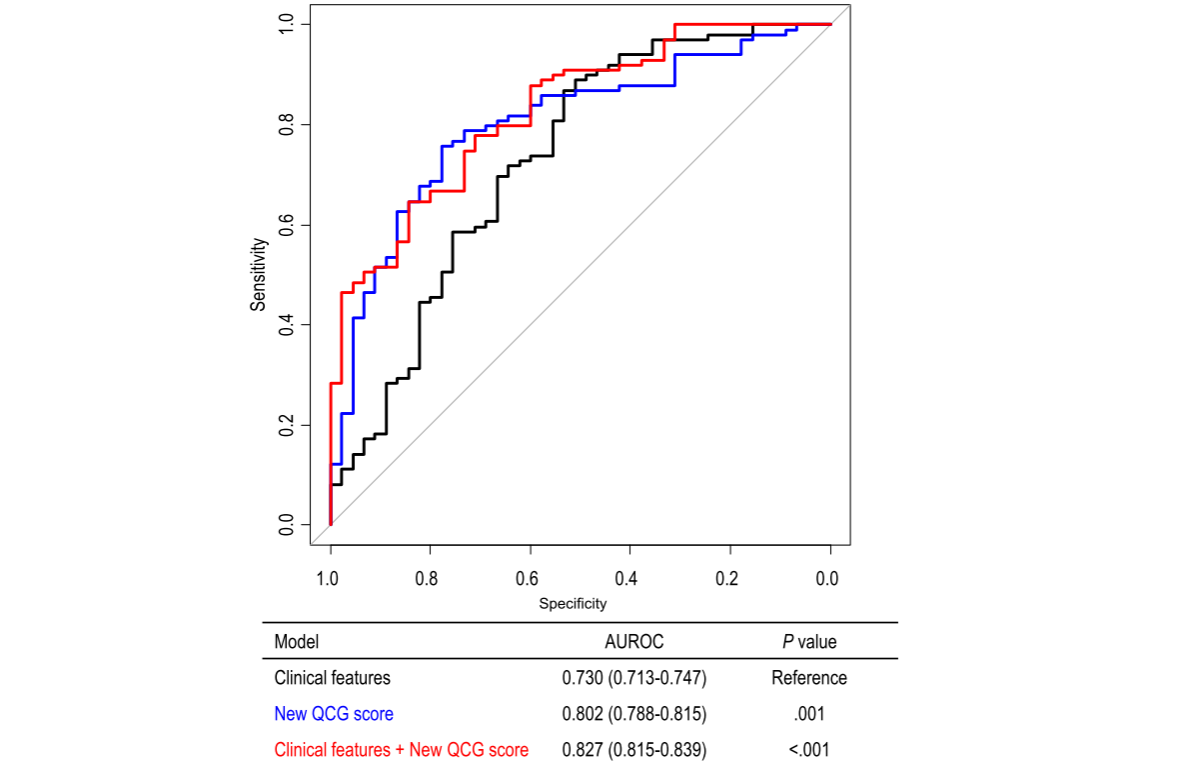
Incremental predictive value of the new quantitative electrocardiography (QCG) score for any obstructive coronary artery disease in the test set. In the test set, the new QCG score showed significantly higher predictive performance for obstructive CAD than that for clinical features, and provided incremental predictive value in combination with clinical features. AUROC: area under the receiver operating characteristic curve.

Based on the above results, a new QCG score for any obstructive CAD more suitable for patients presenting with stable angina was deemed appropriate. The clinical characteristics of the training and test sets are summarized in Table S1 in Multimedia Appendix 1. Although the median age was slightly higher in the training set than in the test set (median 69 years, IQR 60-75 years vs median 65 years, IQR 58-73 years), no significant differences were observed in other clinical features between the training and test sets. The distribution of our new QCG score in the training and test sets is shown in Figure S1 in Multimedia Appendix 2. The new QCG score was significantly higher in patients with obstructive CAD than in those without obstructive CAD. Further, the new QCG score demonstrated improved performance for identifying obstructive CAD (AUROC 0.966 and 0.802 in the training and test sets, respectively) compared to that for the original QCG scores of ACS and myocardial injury (Figure 4). When patients were categorized by the optimal cutoff value of the new QCG score (score of 66 in both training and test sets), the sensitivity, specificity, and accuracy for obstructive CAD were 90.5%, 91.7%, and 90.8% in the training set and 75.8%, 75.6%, and 75.7% in the test set, respectively (Figure 4). In addition, the new QCG score provided incremental predictive value in combination with clinical features (AUROC 0.730 vs 0.827; *P*<.001) (Figure 5).

## Discussion

### Overview

In this study, we evaluated the utility of ECG digital biomarkers developed for acute conditions, known as QCG scores, in the risk stratification of patients with stable angina. Although the discriminative power for the detection of any obstructive CAD was inferior to that for clinical features, CAD-related QCG scores showed independent and incremental predictive value for the presence of 3VD. In addition, to improve the risk stratification of patients with stable angina, we developed a new ECG biomarker for the detection of obstructive CAD in stable angina by using the deep features of the QCG analyzer.

### Challenges in Diagnosing Stable Angina With ECG

ECG has provided valuable insights into the physiological and structural conditions of the heart and has long been used as a primary diagnostic tool for various cardiovascular diseases. In addition to its advantages of low cost, rapidity, and simplicity, ECG acquisition is well standardized and reproducible. However, the human interpretation of ECG images is highly dependent on experience and expertise. Although computer-generated interpretation techniques have been used, they are based on predefined rules and do not capture all of the complex information contained in ECG [12]. In patients with stable angina, the diagnostic value of ECG is often limited because of nonspecific or normal findings in the resting state [3,13,14]. The diagnostic accuracy of ECG in detecting obstructive CAD in the epicardial artery was reportedly low, ranging from 59% to 62% [3]. Nevertheless, it is still possible that there are ECG features relevant to stable angina, but they are so subtle that visual interpretation would inevitably be limited. There is growing evidence that AI techniques using deep-learning convolutional neural networks enable the detection of subtle signals and patterns from ECG that are unrecognizable by the human eye or conventional computer-based analysis [12]. Although they do not fit traditional knowledge, these approaches may allow the prediction of diseases that were previously unpredictable with ECG, such as stable angina. Their usage extends beyond the traditional roles of ECG, such as the identification of the current rhythm, to include novel areas, such as the prediction of subsequent atrial fibrillation events or poor LV ejection fraction [15].

### Potential of AI-ECG Models for Identifying Obstructive CAD in Stable Angina

In terms of CAD, previous studies have demonstrated the feasibility and fine performance of AI-ECG models as a rapid screening tool for ACS in patients presenting with acute chest pain [6-8]. Although various model structures with different AI algorithms have been used, these models have focused on the prediction of ACS. Although it is also conceivable that a well-trained AI-ECG model might be able to detect stable obstructive CAD, there is a paucity of investigations extrapolating the clinical utility of these AI models for identifying obstructive CAD in patients with stable angina. In this study, we hypothesized that predeveloped QCG scores trained on acute-phase patients could also be applied to patients with stable angina. Among 5 QCG scores related to CAD, ACS and myocardial injury QCG scores showed independent associations with the presence of any obstructive CAD. However, their prediction performance was low, and they did not significantly improve the prediction performance over that with clinical features. This is not surprising given that the original QCS scores were derived by a deep-learning process capturing ECG signals of patients in an acute setting. Nevertheless, both scores exhibited moderate performance in predicting obstructive 3VD and showed incremental predictive value in combination with clinical features. Our results suggest that these predeveloped QCG scores also have some potential in capturing ECG changes in patients presenting with stable angina, especially in those with extensive CAD, indicating a higher burden of ischemia, such as 3VD.

### Feasibility of the QCG Analyzer in Stable Angina

Because ECG findings in patients with stable angina are more subtle and nonspecific than those observed in ACS, an accurate prediction of obstructive CAD would be best achieved by a dedicated model for stable angina. Therefore, we proceeded to develop a new QCG score for the presence of obstructive CAD in patients with stable angina based on ECG wave signals vectorized by the QCG analyzer, applying a boosting tree algorithm. The new QCG score performed better than the original QCG scores in predicting obstructive CAD. Notably, the new QCG score showed better performance for the identification of patients with obstructive CAD than the model with conventional clinical risk factors, as well as additive value over that with clinical features.

There have been several reports on AI-ECG models for patients with suspicion of stable CAD. A recent study reported that AI-ECG may predict underlying coronary artery calcification [16]. Another study by Huang et al reported an AI-ECG model for obstructive CAD (defined as >70% diameter stenosis) with an overall accuracy of 90%, which is higher than that in this study (75.7% in the test set) [17]. However, the accuracy in the previous study was only 56% among those without an ECG diagnosis of acute myocardial infarction or ischemia. Additionally, the study population comprised patients with obstructive CAD who underwent percutaneous coronary intervention and control patients without documented or suspected CAD. Therefore, the AI performance was likely overestimated, as the study population comprised extremes (ACS patients with STEMI, who would be easily diagnosed on ECG, and asymptomatic control patients who did not require invasive coronary angiography), and its usefulness may inevitably be limited in clinical practice. In contrast, we enrolled consecutive patients who visited our outpatient clinic with symptoms indicative of stable angina and underwent invasive angiography to confirm the presence of obstructive CAD. Although our study population was limited to a single tertiary center, it is highly representative of our daily clinical practice. Furthermore, the additive value of an AI-ECG analysis of clinical features to detect obstructive CAD could provide valuable insights to clinicians regarding when to consider invasive coronary angiography.

Another study presented a deep-learning model for obstructive CAD (defined as >50% diameter stenosis) in patients with stable angina, similar to that in this study [18]. However, their analysis was based on billing reports and included patients with known CAD (58.3%), including those who underwent coronary revascularization (previous percutaneous intervention [27.4%]; previous coronary artery bypass graft [19%]). Although their model showed higher accuracy for obstructive CAD (overall accuracy of 89.9%) than that in this study, the results may have been affected by high-risk patient profiles, leading to more representative ischemic ECG findings. In this study, we excluded patients with a history of CAD or coronary revascularization, enabling us to demonstrate the feasibility of the QCG analyzer in a population that requires better stratification, and thus benefits from the AI-ECG analysis.

Several AI-ECG models have been developed for commercially available platforms [12]. Although the underlying driving mechanisms may differ between models, they are commonly fed by ECG signals that are preprocessed as 1D or 2D matrix arrays [18]. Therefore, there may be difficulties in applying these AI-ECG models in daily clinical practice, as additional software may be required for input signal transformation. In comparison, the QCG analyzer allows 12-lead ECG image data as input, extracting wave signals and vectorizing them through the initial encoding step [5,6]. Previous reports have validated the consistent performance of the QCG analyzer for printed ECG images and ECG photographs obtained as screenshots from a smartphone [5]. Currently, the QCG analyzer is available as a smartphone app, which can be directly applied on the ECG images obtained by either smartphone cameras or screenshots. Given its user-friendly interface, the QCG analyzer has the potential to be used in both well-equipped hospitals with digital ECGs and more resource-limited settings with paper ECGs. Furthermore, the QCG analyzer can incorporate ECG images obtained from conventional ECG recorders, enabling more effective training in a new disease category. Through further training on a high volume of patients with stable angina, the QCG analyzer can also be an effective screening tool in primary clinics, which initially assess patients with chest pain.

### Limitations

This study has several important limitations. Although we enrolled consecutive patients who visited the outpatient clinic with symptoms indicative of stable angina and underwent invasive coronary angiography, the number of patients was relatively small, limited to a single tertiary center. Because we evaluated the QCG analyzer in symptomatic patients who underwent invasive coronary angiography, our study population may have presented a relatively higher incidence of obstructive CAD compared to previous studies. Furthermore, the performance of the new QCG score, which was derived in this study, was only internally validated. Therefore, the generalizability of our results is limited. Nevertheless, we observed the possibility of detecting obstructive CAD through ECG in patients with stable angina. Therefore, we are planning for further external validation on a larger number of patients to refine the new QCG score and validate it in patient groups with diverse clinical features. We hope we can share the results in the near future.

### Conclusions

As a quantitative AI-ECG algorithm, the QCG analyzer shows the feasibility of predicting obstructive CAD in patients with stable angina. Although predeveloped QCG scores for CAD-related conditions showed limited performance for the detection of obstructive CAD in stable angina, they still demonstrated independent and incremental predictive value for the presence of 3VD. Furthermore, we developed a new QCG score by using the ECG wave signals vectorized by the QCG analyzer, which outperformed the conventional model with clinical features. With further expanded training on stable angina, the QCG analyzer could be a more accurate and cost-effective AI tool for screening obstructive CAD in these patients.

## Appendix

Multimedia Appendix 1 Patient characteristics in training and test sets.

Multimedia Appendix 2 Distribution of the new quantitative electrocardiography score.

## Abbreviations

ACS: acute coronary syndrome
AI: artificial intelligence
AUROC: area under the receiver operating characteristic curve
CAD: coronary artery disease
ECG: electrocardiography
LM: left main
LV: left ventricular
OR: odds ratio
QCG: quantitative electrocardiography
RCA: right coronary artery
SNUBH: Seoul National University Bundang Hospital
STEMI: ST-elevation myocardial infarction
VD: vessel with obstructive CAD

## Data Availability

Data cannot be made publicly available due to ethical restrictions set by the institutional review board of Seoul National University Bundang Hospital; that is, public availability would compromise patient confidentiality and participant privacy. Please contact the corresponding authors (yeonyeeyoon@snubh.org or cho_y@snubh.org) to request the anonymized data set.
