# Supplementary material for: Feasibility of Artificial Intelligence–Based Electrocardiography Analysis for the Prediction of Obstructive Coronary Artery Disease in Patients With Stable Angina: Validation Study
Source: JMIR Cardio. 2023 May 2;7:e44791. doi: 10.2196/44791 (PMC10189614; doi:10.2196/44791)
Supplement: Multimedia Appendix 1 [file cardio_v7i1e44791_app1.pdf]

Table S1. Patient characteristics in Training and Test sets

Variables[a]	Training set (n=579)	Test set (n=144)	*P*
**Demographics**			
Age, years	69 (60-75)	65 (58-73)	.003
Male	381 (65.8)	89 (61.8)	.42
BMI, kg/m^2	25.2 (23.4-27.1)	25.0 (23.2-27.2)	.80
**Clinical features**			
**Symptoms**			.42
Typical chest pain	392 (67.7)	94 (65.3)	
Atypical chest pain	132 (22.8)	31 (21.5)	
Angina equivalent	55 (9.5)	19 (13.2)	
Hypertension	370 (63.9)	89 (61.8)	.71
Diabetes mellitus	180 (31.1)	41 (28.5)	.61
Dyslipidemia	204 (35.2)	41 (28.5)	.15
Stroke	35 (6.0)	6 (4.2)	.50
Family history of CAD	45 (7.8)	11 (7.6)	.55
Smoking	54 (9.3)	14 (9.7)	.99
**Invasive coronary angiography**			
**Obstructive CAD**			
Left main	45 (7.8)	11 (7.6)	.97
LAD	318 (54.9)	84 (58.3)	.52
LCX	173 (29.9)	45 (31.2)	.83
RCA	201 (34.7)	47 (32.6)	.71

Any obstructive CAD	398 (68.7)	99 (68.8)	.99
3-vessel obstructive CAD	103 (17.8)	29 (20.1)	.59

[a]Values are given as numbers (percentage) or median (IQR)
BMI, body mass index; CAD, coronary artery disease; LAD, left anterior descending artery; LCX, left circumflex artery; PCI, percutaneous coronary intervention; RCA, right coronary artery
