# Supplementary material for: Feasibility of Artificial Intelligence–Based Electrocardiography Analysis for the Prediction of Obstructive Coronary Artery Disease in Patients With Stable Angina: Validation Study
Source: JMIR Cardio. 2023 May 2;7:e44791. doi: 10.2196/44791 (PMC10189614; doi:10.2196/44791)

Figure S1. Distribution of the new QCG score. CAD, coronary artery disease; QCG, quantitative electrocardiography

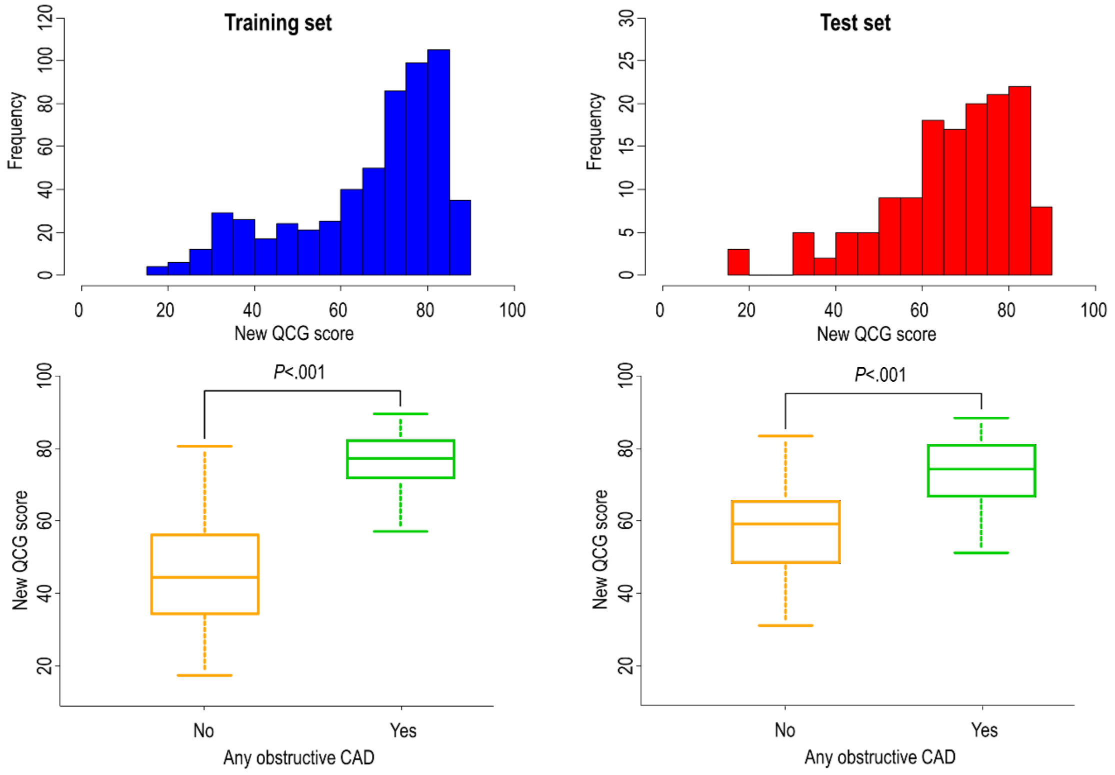

Supplement: Multimedia Appendix 2 [file cardio_v7i1e44791_app2.pdf]
